# Facet joint hypertrophy is a misnomer: A retrospective study

**DOI:** 10.1097/MD.0000000000011090

**Published:** 2018-06-15

**Authors:** Sang Joon An, Mi Sook Seo, Soo Il Choi, Tae-Ha Lim, So Jin Shin, Keum Nae Kang, Young Uk Kim

**Affiliations:** a Department of Neurology; b Department of Anesthesiology and Pain Medicine, Catholic Kwandong University of Korea College of Medicine, International ST. Mary‘s Hospital, Incheon; c Department of Anesthesiology and Pain Medicine, Eulji General Hospital, Eulji University College of Medicine; d Department of Anesthesiology and Pain Medicine, National Police Hospital, Seoul, Republic of Korea.

**Keywords:** facet joint cross-sectional area, facet joint hypertrophy, facet joint thickness, lumbar spinal canal stenosis

## Abstract

One of the major causes of lumbar spinal canal stenosis (LSCS) has been considered facet joint hypertrophy (FJH). However, a previous study asserted that “FJH” is a misnomer because common facet joints are no smaller than degenerative facet joints; however, this hypothesis has not been effectively demonstrated. Therefore, in order to verify that FJH is a misnomer in patients with LSCS, we devised new morphological parameters that we called facet joint thickness (FJT) and facet joint cross-sectional area (FJA).

We collected FJT and FJA data from 114 patients with LSCS. A total of 86 control subjects underwent lumbar magnetic resonance imaging (MRI) as part of routine medical examinations, and axial T2-weighted MRI images were obtained from all participants. We measured FJT by drawing a line along the facet area and then measuring the narrowest point at L4-L5. We measured FJA as the whole cross-sectional area of the facet joint at the stenotic L4-L5 level.

The average FJT was 1.60 ± 0.36 mm in the control group and 1.11 ± 0.32 mm in the LSCS group. The average FJA was 14.46 ± 5.17 mm^2^ in the control group and 9.31 ± 3.47 mm^2^ in the LSCS group. Patients with LSCS had significantly lower FJTs (*P* < .001) and FJAs (*P* < .001).

FJH, a misnomer, should be renamed facet joint area narrowing. Using this terminology would eliminate confusion in descriptions of the facet joint.

## 1 Introduction

Lumbar spinal canal stenosis (LSCS) results from degenerative changes in the spinal canal and is one of the most common spinal disorders in elderly individuals.^[1–3]^ It is characterized by narrowing of the spinal canal and is caused by hypertrophy of the ligamentum flavum, mechanical compression of the lumbar spinal nerve roots, and disc herniation combined with osteophytes.^[4,5]^ Facet joint hypertrophy (FJH) is considered another major cause of LSCS.^[6]^ The facet joints play an important role in maintaining the stability of the spinal column.^[7]^ Furthermore, changes in the mechanical facet joint environment have been associated with degeneration and osteoarthritis, either of which could eventually lead to LSCS.^[7,8]^ The spinal canal can be narrowed by characteristic changes in the facet joints such as hypertrophy of articular processes, synovial cysts, or osteoarthritis.^[9,10]^

However, Barry and Livesley^[6]^ asserted that “FJH” is a misnomer because normal facet joints are no smaller than degenerative facet joints. Their assertion has been hypothesized but has not been effectively demonstrated. Therefore, in order to verify that facet join hypertrophy is a misnomer in LSCS patients, we devised 2 new morphological parameters, facet joint thickness (FJT) and facet joint area (FJA). FJT and FJA have not yet been evaluated for their associations with LSCS. We hypothesized that both would be important morphologic parameters for identifying facet joints.

## 2 Materials and methods

### 2.1 Patients

The Catholic Kwandong University College of Medicine, Republic of Korea, Institutional Review Board (IRB) reviewed and approved the research project (IRB protocol number: IS17RISI0032). We retrospectively reviewed patients who had visited our pain clinic between March 2014 and June 2017 and had been diagnosed with LSCS. We included patients over age 60 if they had clinical manifestations compatible with LSCS (such as low back pain and/or neurogenic intermittent claudication), the most stenosis at L4-L5, and MRI performed within 12 months of the diagnosis that was available for review. We excluded patients if they had a history of previous lumbar surgery or spinal injury, congenital spine defects, history of spinal interventions such as kyphoplasty, or any anatomic anomalies.

We enrolled a total of 114 patients after the LSCS diagnosis was confirmed by 2 experienced, board-certified neuroradiologists. The measurement analysis and data collection was performed in a double-blind fashion. In the LSCS group, there were 28 (24.56%) males and 86 (75.44%) females with a mean age of 68.15 ± 5.66 years (range: 60–87 years; Table 1). To compare the FJAs and FJTs between patients with and without LSCS, we enrolled a group of control patients who had undergone lumbar MRI as part of routine medical examinations and who had no LSCS-related symptoms. The control group consisted of 86 participants [31 males (36.05%) and 55 females (63.95%)] with a mean age of 69.51 ± 7.72 years (range: 60–89 years; Table 1). We also examined the FJAs and FJTs in the control group at the L4-L5 facet joint level.

**Table 1:**
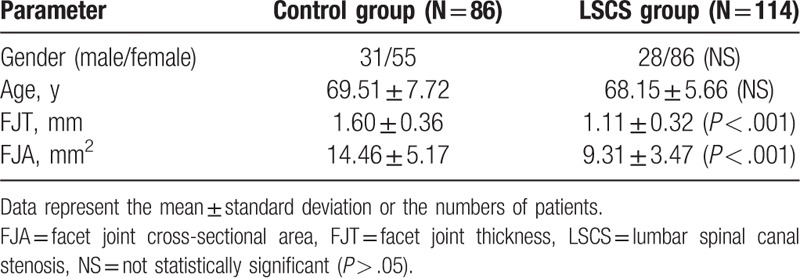
Comparison of the characteristics of control and LSCS groups.

### 2.2 Imaging parameters

The MRI examinations had been performed with 3T scanners (Magnetom Skyra, Sonata, Biograph, Avanto, Siemens Healthcare, and Philips Ingenia [R4], Philips Medical Systems, Best, The Netherlands), and axial T2-weighted images with 4 mm thick slices had been obtained. The following other parameters were used as well: 0.4 mm intersection gap, 3000 ms/90 ms repetition time/echo time, 180 × 180 cm field of view, 448 × 270 matrix, and 15 echo train length (ETL). Sagittal T2-weighted images with 4 mm slice thickness were obtained. The following other parameters were used: 0.4 mm intersection gap, 2700 ms/95 ms repetition time)/echo time, 300 × 300 cm field of view, 358 × 512 matrix, and 15 ETL.

### 2.3 Image analysis

**Figure 1:**
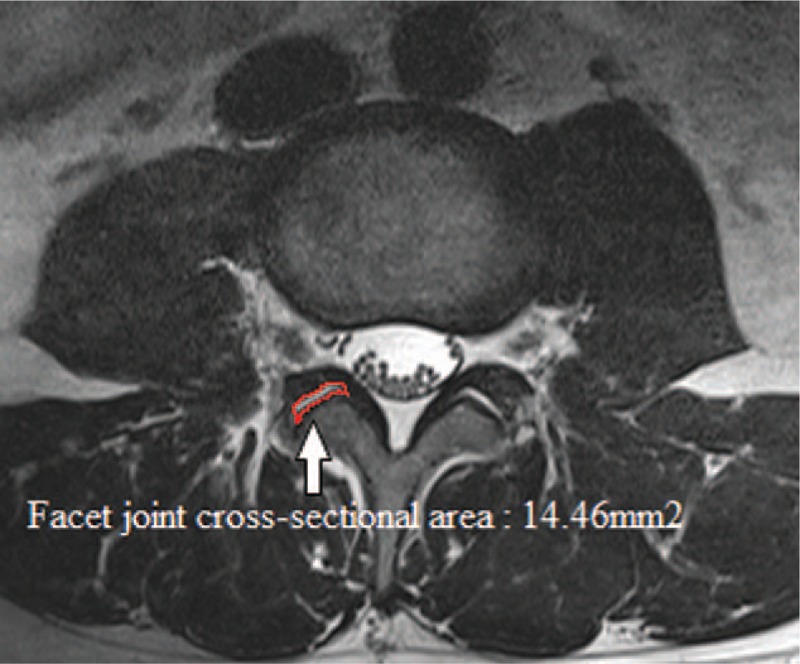
Measurement of the facet joint area on MRI at the L4-L5 level.

**Figure 2:**
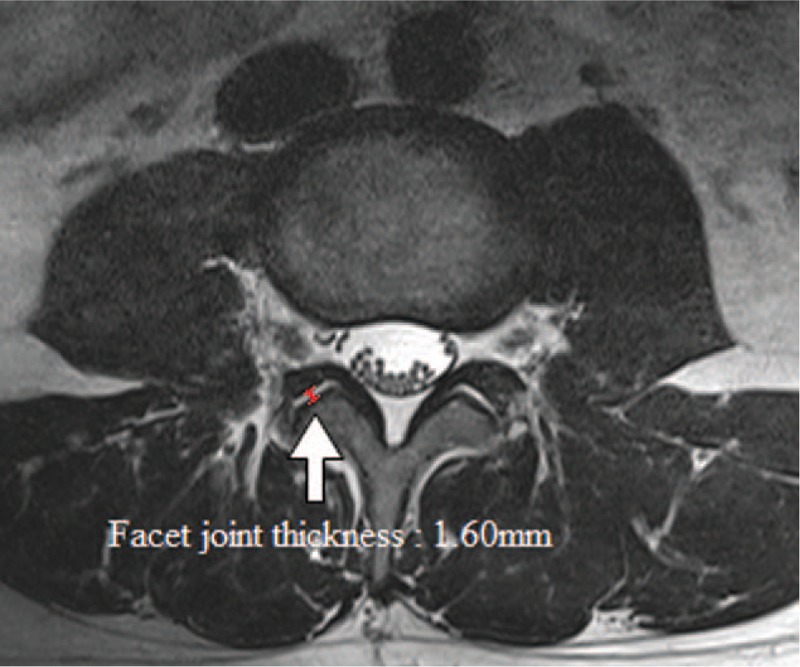
Measurement of the facet joint thickness on MRI at the L4-L5 level.

The axial T2-weighted MR images had been acquired at the facet joint level for individual patients. We used a picture archiving and communications system to measure the FJAs and FJTs at the L4-L5 facet joints on MRI. We measured the FJA as the cross-sectional area by outlining the facet joint at L4-L5 (Fig. 1) and the FJT by drawing a line along the joint and then measuring the narrowest point at L4-L5 (Fig. 2).

### 2.4 Statistical analysis

We analyzed the data as means ± standard deviations (SD), and we used unpaired *t* tests to compare the FJTs and FJAs between the control and LSCS groups; we set significance at *P* < .05. We also analyzed the relationships between the FJT, the FJA, and age-related changes using 1-way analysis of variance (ANOVA). We performed all statistical analyses with SPSS for Windows version 21 (IBM SPSS, IBM Corp., Armonk, NY).

## 3 Results

The demographic data were not significantly different between the groups (Table 1). The average FJTs were 1.60 ± 0.36 mm in the control group and 1.11 ± 0.32 mm in the LSCS group, and the average FJAs were 14.46 ± 5.17 mm^2^ in the control group and 9.31 ± 3.47 mm^2^ in the LSCS group. The patients with LSCS had significantly lower FJTs (*P* < .001) and narrower FJAs (*P* < .001; see Table 1). The mean FJTs and FJAs in the control group were 1.66 ± 0.37 mm and 13.33 ± 5.39 mm^2^ in subjects aged 60 to 69 years, 1.48 ± 0.29 mm and 15.78 ± 3.44 mm^2^ in those aged 70 to 79 years, and 1.73 ± 0.49 mm and 16.34 ± 7.77 mm^2^ in those aged 80 to 89 years (Table 2). In the control group, we found no statistically significant relationships between the FJT (F = 2.908; df = 2; *P* = .060), the FJA (F = 2.777; df = 2; *P* = .068), and age-related changes on 1-way ANOVA. The mean FJTs and FJAs in the LSCS group were 1.13 ± 0.35 mm and 8.97 ± 3.61 mm^2^ in those aged 60 to 69 years, 1.12 ± 0.24 mm and 9.91 ± 2.96 mm^2^ in those aged 70 to 79 years, and 0.86 ± 0.26 mm and 10.72 ± 3.89 mm^2^ in those aged 80 to 89 years (Table 3). In the LSCS group, no statistically significant relationships were evident between the FJT (F = 1.967; df = 2; *P* = .145), the FJA (F = 1.338; df = 2; *P* = .266), and age-related changes.

**Table 2:**
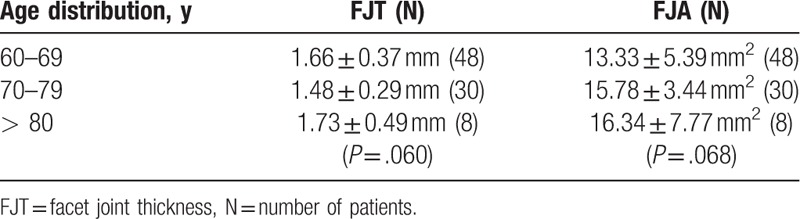
Age distribution of patients with mean FJT and FJA in the control group.

**Table 3:**
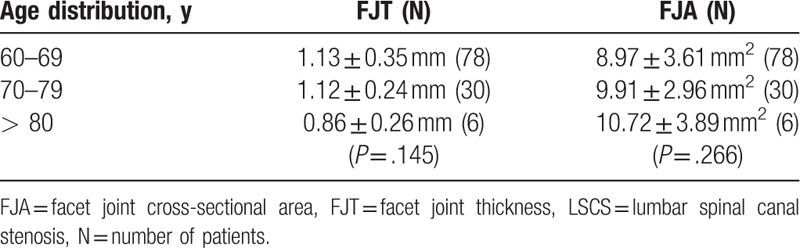
Age distribution of patients with mean FJT and FJA in the LSCS group.

## 4 Discussion

LSCS is a common pathologic condition in elderly individuals that causes intermittent neurogenic claudication and low back or buttock pain^[11]^; it results from a combination of pathogenic factors, including hypertrophy of the ligamentum flavum, a decrease in the area of the cauda equina, loss of intervertebral disk height, and hypertrophy of the facet joints.^[12]^ In addition, overgrowth of the facet joint capsule can lead to LSCS.^[5,13]^ Therefore, FJH has been considered a major cause in the development of LSCS. Degenerative changes in facet joints also include subchondral sclerosis, osteophytosis, joint surface irregularity, and apophyseal hypertrophy.^[14–16]^ Many previous studies have investigated facet joints. Little et al^[14]^ investigated the reliability of a 5-point scale that grades the severity of degenerative facet joint changes: Grade 0 = absence of joint degeneration at the center of the radiograph, I = questionable osteophytes on the superior joint margin, II = subchondral sclerosis and definite joint osteophytes, III: subchondral sclerosis, some joint irregularity, and moderate osteophytes, and IV = severe sclerosis, irregularity of the articular joint surfaces, and many osteophytes. The authors asserted that this grading system may be useful for assessing facet joint osteoarthritis.^[14]^ Takashima et al^[17]^ demonstrated that facet joints are important for the segmentation and stability of the lumbar spinal column and that they possess articular cartilage. Therefore, osteoarthritis occurs in facet joints as it does in other synovial joints. Bajek et al^[18]^ explained that osteophyte formation in the lumbar spine is an attempt to stabilize an unstable segment; this mechanism ultimately leads to FJH. Disc degeneration may also increase the stressful force on the facet joints.^[19]^

However, Barry and Livesley^[6]^ reported that “FJH” is a misnomer because normal facet joints are no smaller than degenerate facet joints. These authors also contended that there is no clear definition in the literature regarding lumbar FJH.^[6,20]^ But this hypothesis has not been confirmed. Therefore, in order to verify that FJH is a misnomer in patients with LSCS, we devised new morphological parameters we called the FJT and FJA. We believe that the FJT and FJA are the precise, objective measurement parameters to correct the mistaken terminology, and our results show that the patients with LSCS had significantly lower FJAs and narrower FJTs than did control subjects. It may be that any degenerative facet joint changes could be termed hypertrophic, but this imprecise term is not supported by the results of this study; in the present study, we measured both FJT and FJA. Although FJT can reflect significant facet joint space narrowing, the shape of the facet joint is not always regular and the direction of the axis of the facet surface cannot be determined.^[21]^ To supplement these measurement errors, we also measured the whole cross-sectional area of the facet joint. Analyzing FJA is beneficial for comparing cartilage degeneration with facet joint structure.^[21]^ Biomechanically, the function of facet joints is to limit and guide movement of that spinal column.^[7]^ Our interpretation of these associations is that facet joint narrowing may be related to extensive loading during motion, which might contribute to facet joint osteoarthritis.^[21,22]^ The process of facet joint narrowing begins with stress during lumbar flexion and rotation. These mechanical stressors put force on the facet joints, which leads to a high degree of abrasion,^[23,24]^ and this etiology may alter the morphologic features of the facet joint area. If this is accurate, what is the way to correct this misnomer? Previously, authors have concluded that osteophytes and hypertrophy of the superior articular process were the main factors of facet joint narrowing.^[25]^ FJH in the LSCS refers to hypertrophy in the superior articular process and may be associated with facet joint narrowing. For simplicity, facet joint changes could be referred to as “facet joint narrowing.” Using this terminology, descriptions of facet joints would not be confused with superior articular process hypertrophy.

Farrell et al^[26]^ described the morphological patterns of the zygapophyseal joint. These cross-sectional areas were analyzed from cadaveric hemi-spines. Simon et al^[21]^ described the facet joint space width by measuring the cross-sectional area of the facet joint space using 3D computed tomography.

In this study, we measured the FJT and FJA from MRI images. Although MRI is the most important modality for characterizing LSCS and facet joint lesions,^[17]^ there are no previous reports of an association between LSCS and facet joints as a morphologic parameter on MRI. Therefore, we used MRI to compare the FJTs and FJAs between patients with LCSS and healthy controls; to our knowledge, these measurements have not been previously reported. This study only included individuals > 60 years old because previous studies have demonstrated that articular cartilage thinning, subarticular cortical bone hypertrophy, and narrowing of the facet joint gap are observed age-related changes.^[21]^

This study has some limitations. First, although we measured the FJA and FJT in axial T2 images at the L4-5 facet joint, there may be errors associated with measuring these on MRI because these axial images may not be homogeneous due to differences in the cutting angle of the MRI resulting from individual anatomic variations and technical problems; in addition, the 4.0 mm slice of axial T2-weighted MR image is also thicker than an ideal slice. Second, the small sample sizes in some age groups can lead to less than ideal data analysis. Baseline demographic data of the patient population such as body weight and height vary widely. Third, we measured FJT at the narrowest distances between the inferior and superior facet joint surfaces; therefore, we could not estimate the cartilage widths at individual facet joints using this technique. Fourth, several different parameters are known to effectively discriminate LSCS, such as morphological grading and analysis of cauda equina.^[27,28]^ However, this study only investigated lumbar facet joint. Finally, another limitation of this study is its retrospective nature. Prospective researches are needed to validate and repeat our results. Despite these limitations, this is the first objective study to verify that FJH is a misnomer in patients with LSCS, and these results may be valuable information to analyze further exact diagnostic terminology when assessing LSCS.

## 5 Conclusion

Our results demonstrate that FJH is a misnomer, and we suggest that it be renamed facet joint area narrowing. We believe that this renaming will help physicians in their evaluations of patients with LSCS. We also hope that pain physicians will no longer use the term “facet joint hypertrophy.”

## Acknowledgment

We gratefully acknowledge Gyung-A Chun for support with image management.

## Author contributions

**Conceptualization:** Young Uk Kim.

**Data curation:** Young Uk Kim.

**Formal analysis:** Soo Il Choi, Young Uk Kim.

**Investigation:** Sang Joon An.

**Methodology:** Sang Joon An, Mi Sook Seo.

**Resources:** Sang Joon An.

**Supervision:** Tae-Ha Lim, So Jin Shin.

**Validation:** Young Uk Kim.

**Visualization:** Young Uk Kim.

**Writing – original draft:** Sang Joon An, Young Uk Kim.

**Writing – review & editing:** Mi Sook Seo, Keum Nae Kang.
